# Mental template matching is a potential cultural transmission mechanism for New Caledonian crow tool manufacturing traditions

**DOI:** 10.1038/s41598-018-27405-1

**Published:** 2018-06-28

**Authors:** S. A. Jelbert, R. J. Hosking, A. H. Taylor, R. D. Gray

**Affiliations:** 1 Department of Psychology, University of Cambridge, Cambridge, CB2 3EB UK; 2 School of Psychology, University of Auckland, Auckland, 1010 New Zealand; 3 Center for e-Research, University of Auckland, Auckland, 1010 New Zealand; 4 Max Planck Institute for the Science of Human History, Jena, 07745 Germany; 5 Research School of the Social Sciences, Australian National University, Canberra, 2601 Australia

## Abstract

Cumulative cultural evolution occurs when social traditions accumulate improvements over time. In humans cumulative cultural evolution is thought to depend on a unique suite of cognitive abilities, including teaching, language and imitation. Tool-making New Caledonian crows show some hallmarks of cumulative culture; but this claim is contentious, in part because these birds do not appear to imitate. One alternative hypothesis is that crows’ tool designs could be culturally transmitted through a process of *mental template matching*. That is, individuals could use or observe conspecifics’ tools, form a mental template of a particular tool design, and then reproduce this in their own manufacture – a process analogous to birdsong learning. Here, we provide the first evidence supporting this hypothesis, by demonstrating that New Caledonian crows have the cognitive capacity for mental template matching. Using a novel manufacture paradigm, crows were first trained to drop paper into a vending machine to retrieve rewards. They later learnt that only items of a particular size (large or small templates) were rewarded. At test, despite being rewarded at random, and with no physical templates present, crows manufactured items that were more similar in size to previously rewarded, than unrewarded, templates. Our results provide the first evidence that this cognitive ability may underpin the transmission of New Caledonian crows’ natural tool designs.

## Introduction

Cultural traditions are common in the animal kingdom^1–3^, but cumulative cultural evolution is rare^4^.

Despite decades of study of animal traditions (group-typical behaviour patterns that rely on socially transmitted information^5^) – such as the iconic sweet potato washing by macaques or milk bottle opening by tits^6,7^ – there is minimal evidence that these, or any other, animal traditions have evolved and accumulated improvements over time (cumulative cultural evolution)^3,8^. This contrasts sharply with findings from the human archaeological record, where clear indications of cumulative culture are present from at least 100,000 years ago^9^. Moreover, technological transitions from Oldowan flake-based tools, to more standardised Acheulean bifacial hand-axes, suggest that the cumulative evolution of technology could have begun as early as 1.6 million years ago in our lineage^10^.

Several researchers have argued that a suite of adaptations were required to enable this ratchetting up of technologies and traditions, including our uniquely human capacities for teaching, language and imitation^8,11–13^. However, social learning is not just underpinned by the copying of actions. It is defined as learning that is “influenced by observation of, or interaction with another animal *or its products*”^14,15^ (our emphasis). It has been argued that emulative mechanisms – learning from observing the end-products, rather than the actions which produced the products – can produce only low-fidelity copying, insufficient to support cumulative cultural change^8^. However, it remains possible that copying end-products might offer an alternative route towards cumulative cultural evolution in some situations^16^. In the laboratory, transmission chain studies demonstrate that both adults^17,18^ and children^19^ can replicate or iteratively improve on the design of manufactured artefacts when provided only with end-products to copy (allowing for emulation), without teaching, language or the opportunity to observe the manufacture process (i.e. without imitation). Though subjects are provided with spoken and written instructions as to the goal of the task. This demonstrates that action information is not always necessary for cultural transmission, and that, at least in certain artificial settings, cumulative culture can emerge in humans through emulative social learning processes, focused only on learning from *products*.

Among non-human animals, tool-making New Caledonian crows are remarkable in that they produce tools which show some of the hallmarks of cumulative cultural evolution^20^. Across the island of Grande Terre, New Caledonian crows manufacture basic stick tools, hooked stick tools^21^ and barbed tools torn from the leaves of pandanus plants^22^. They routinely manufacture at least three distinct pandanus tool designs in the wild, including wide (short, wide leaf sections, ca. 15 by 0.5 cm), narrow (long, thin leaf sections, ca. 23 by 0.25 cm), and stepped designs, where stepped tools taper from a wide base to a narrow working tip in a series of rips and cuts. The specific tool designs made in different areas do not have obvious ecological correlates, and have persisted for at least several decades (at least pre-2000 to present)^20^, suggesting high-fidelity transmission. The functionality of different pandanus tool designs is not yet well understood; however, their geographic distribution raises the possibility that stepped tools represent modifications made to the simpler wide design. Thus, New Caledonian crows may possess a material culture that has incorporated incremental changes over time^20^.

At present, whether New Caledonian crow tool designs are culturally transmitted, and have evolved over time, remains contentious^23^. In part this is due to an absence of evidence, in this species, for the types of sophisticated social learning mechanisms thought to be necessary for such behaviour. Specifically, New Caledonian crows do not appear to imitate^24,25^; nor do these birds teach or possess language^26,27^. In a social learning experiment, captive New Caledonian crows exhibited stimulus enhancement, but no other social learning mechanisms, when retrieving food from a puzzle box in the presence of trained demonstrators^24^. They do not appear to closely observe the process of tool manufacture in the wild^26^ and experiments in captivity suggest they may have poor social cognition^28^. However, one hypothesis is that New Caledonian crow tool designs could be culturally transmitted – without teaching, language or imitation – through a form of end-state emulation, termed *mental template matching*^24,26^. Under the mental template matching hypothesis, New Caledonian crow tool designs could be passed on to subsequent generations if an individual used or observed the *products of tool manufacture* (such as their parents’ tools), formed a mental template of this type of tool design (a mental representation of some or all of the tool’s properties), and then reproduced this template in their own manufacture. This mechanism can be considered analogous to avian song learning, in which juveniles first acquire a song template from listening to conspecifics, and then later adjust their own vocalisations until they match that of the memorised template^29^. Significantly, the formation of a *mental* template would enable a bird to produce standardised tools without the requirement that existing tools are visible during manufacture. Most importantly, an improvement made by a crow during its lifetime could become part of the template learnt by subsequent generations, leading to an increase in tool complexity over time. Mental template matching is therefore a specific type of end-state emulation that could potentially allow for cumulative cultural change in the design of material artefacts.

Here, we provide the first test of the template matching hypothesis in New Caledonian crows. This hypothesis makes a clear prediction: that New Caledonian crows have the cognitive capacity to manufacture items that are similar to previously experienced templates. We developed an arbitrary manufacture task that mirrored pandanus tool manufacture, in that it required the ripping of material in order to gain food. However, instead of pandanus leaves, we used an unfamiliar manufacture material (card). By requiring crows to use this man-made material to create items that take different shapes than pandanus tools, our task had sufficient novelty to prevent the crows from transferring learnt rules formed during their prior tool manufacture experience in the wild. Eight New Caledonian crows learnt to drop squares of white paper into a vending machine to receive rewards. They later learnt that only pieces of card of a specific size (either large templates: 40 × 60 mm or small templates: 15 × 25 mm) were rewarded. Having learnt which template was successful, birds received manufacture probe trials where very large sheets of card were provided, from which they could rip sections to drop into the vending machine – a form of manufacture by subtraction^30^. After manufacturing 20 pieces, they were trained that the alternative size (large or small) was rewarded, and the manufacture test was repeated. Conditions were counterbalanced across birds. No templates were present during manufacture test trials, and, to exclude the possibility of operant conditioning or trial-and-error learning during these trials, crows were rewarded at random for 50% of the items they manufactured and dropped into the vending machine. Our experiment, therefore, required crows to make either large or small card pieces at test, without reference to a card template they could see, and without differential reinforcement during the test for making pieces of a particular size.

## Results

**Figure 1:**
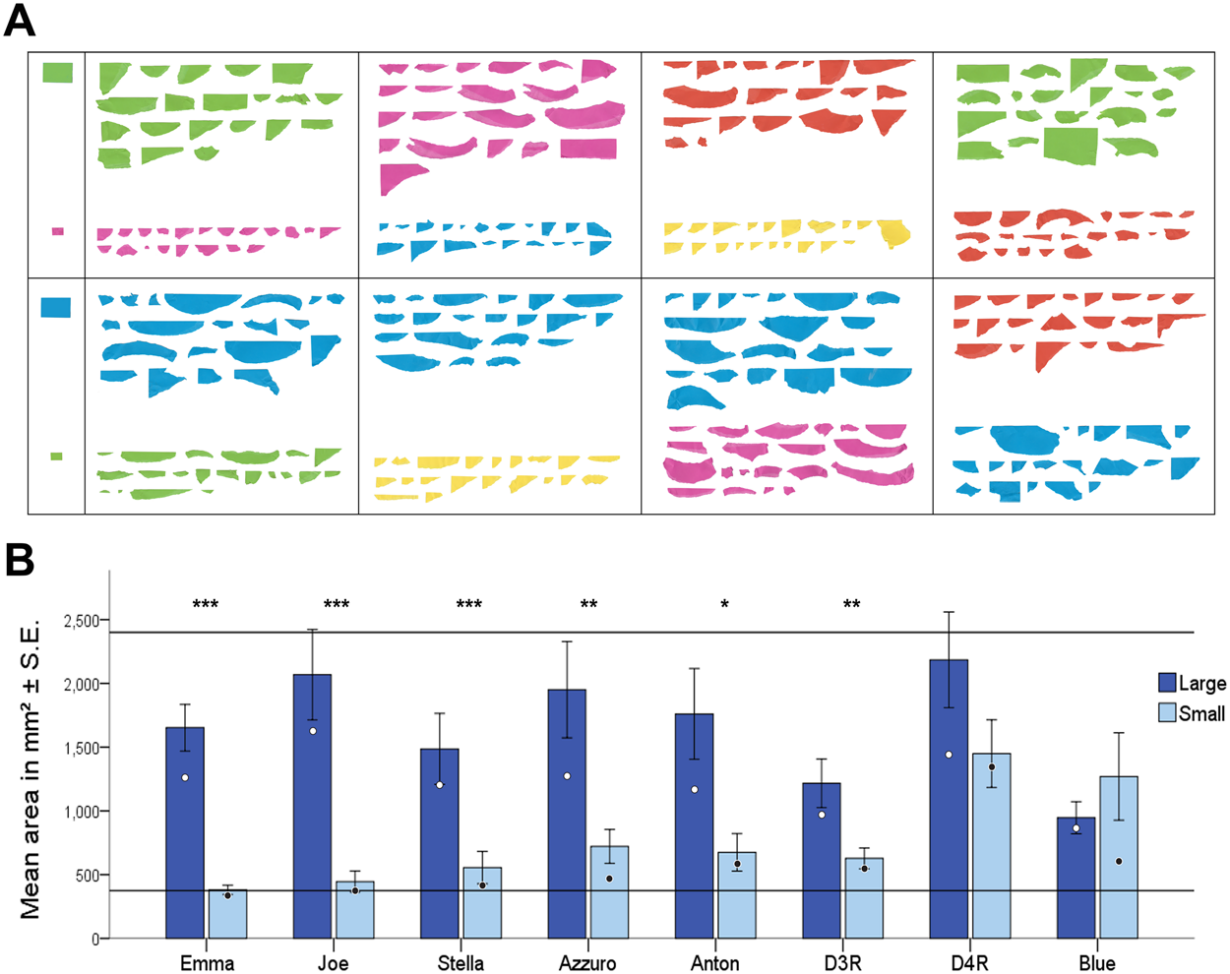
The pieces manufactured by each bird in each condition. (**A**) Scanned images of all pieces ripped by each bird after learning that ‘small’ or ‘large’ templates were rewarded, in the order in which they were produced. Example templates are provided on the left. Condition order was counterbalanced. Bird names (from L-R) top row: Emma, Joe, Stella, Azzuro (all adults); bottom row: Anton, D3R, D4R, Blue (all juveniles less than 2 years old), order as in Panel (B) Panel B: The mean area of pieces ripped by each bird after learning that ‘small’ or ‘large’ templates were rewarded. Circles denote the median values. Horizontal lines indicate the area of the small and large templates. Mann-Whitney U-tests: ***p < 0.001, **p < 0.01, *p < 0.05.

We observed that New Caledonian crows manufactured differently sized card pieces after learning that either large or small templates were rewarded. The mean area of manufactured pieces was 2.47 times larger when birds had learnt that a large, rather than small, template was rewarded (LMM: *p* < 0.001). Individually, six of the eight birds manufactured significantly differently sized pieces in the two conditions (4 adults, 2 juveniles; Mann Whitney U-tests: *p* < 0.05, Fig. 1). The two birds that did not were both juveniles. There was a significant difference in size over time in both conditions; in the large condition, birds manufactured larger pieces in later trials (GLMM: p = 0.0004, Supplementary Figure S1) and, in the small condition, they manufactured smaller pieces in later trials (GLMM: p = 0.05 Supplementary Figure S1). This is consistent with subjects improving their manufacture technique over time, as occurs in the wild^26^. It is not consistent with trial-and-error learning or operant conditioning during these manufacture test trials, because birds were rewarded at random. Birds had been presented with the templates shortly before the manufacture trials, but no templates were present during the manufacture trials. Thus, at test the size of the manufactured pieces could only have been influenced by the crows’ prior experience: learning which of two differently sized templates was rewarded during the earlier object choice task.

The behaviour of one bird (Emma, an adult female) provides compelling evidence that this species has the ability to manufacture items that match the absolute, not just relative, sizes of rewarded templates. On a number of trials, after detaching a section of card, Emma modified the size of the detached piece before dropping it into the vending machine (see the Supplementary Video S1 for an example of this behaviour). This occurred on 5/20 trials in the large condition, and 1/20 in the small condition. In all cases, modifications were made to pieces that were longer than the template, and, following modification, all pieces became more similar in length to the rewarded template. Modifications were made in the small condition by a further 3 birds (Azzuro: 2, Stella: 1, Joe: 1), but only Emma reduced the size of overly large pieces in the large condition. In line with this behaviour, Emma’s pieces were particularly accurate. Based on their length and width, all pieces except one (39/40) were more similar to the rewarded than the unrewarded template (Supplementary Figure S2).

## Discussion

Our results provide the first evidence to suggest that New Caledonian crows have the cognitive capacity to manufacture objects from a mental template. The New Caledonian crows tested here manufactured items that matched the relative size of the previously rewarded templates, without being rewarded for doing so during manufacture test trials and without templates being present at the time of manufacture. One bird in particular, Emma, manufactured pieces that were highly similar to each template, and made secondary modifications to reduce the length of overly large pieces. This strongly suggests that this crow possessed a capacity to remember and reproduce the absolute, not just relative, size of rewarded templates.

Alternative explanations to the crows using mental representations of the different, previously experienced card designs to drive manufacture are ruled out by the design of our study. Crows were not rewarded for manufacturing card of different sizes during training. In fact, crows had only been rewarded for ripping pieces of paper or card *irrespective of size* during training and so would be predicted to rip pieces of card of any size if they simply transferred learnt rules from training to test. Instead, crows clearly used their prior experience of *choosing* card of a particular size to then guide subsequent card *manufacture*, despite no small or large card templates being visible for crows to base manufacture off and the apparatus being identical across conditions. Even during the test, due to us rewarding 50% of card manufacture trials, irrespective of the size made, there was no differential reinforcement that the crows could have used to guide their tool manufacture. Thus, the only way that crows could have made a card template of the correct size was if they had a mental representation of its size, there were simply no physical cues available to guide them.

Our results provide evidence for one of the key predictions of the mental template hypothesis, namely that New Caledonian crows have the cognitive capacity to manufacture items that are similar to previously experienced templates. While there are clearly several other predictions of this hypothesis that require testing, given the results here, we argue that mental template matching is now the leading hypothesis to explain why New Caledonian crow tools show some of the hallmarks of cumulative cultural evolution. Other hypotheses, such as language, teaching, and imitation, can be ruled out due to past work establishing that New Caledonian crows do not have these abilities. In contrast, the mental template matching hypothesis is supported by the results here and recent work showing that humans can iteratively improve on the design of manufactured artefacts such as constructing paper planes^18^ or baskets to transport rice^17^, when *copying solely the products of social learning*, rather than observing an interaction between another social agent and the product. Finally, the mental template matching hypothesis fits well with this species’ ecology. The tendency to acquire basic stick tool manufacture is widespread, develops early, and appears to have a genetic basis in these crows^31^. In contrast, pandanus tool manufacture is not universal, and, when it does occur, adult-like tool manufacture develops slowly over the first year of life^26^. During this time, juveniles associate closely with their parents^32^, regularly borrowing their parents’ tools and using them to acquire food^26^. Thus, juveniles have ample opportunities to form a mental template of a particular tool design in the wild from both observing and using the tools of their parents. This template allows these crows to recreate this tool even when neither parents nor the parents’ tools are within sight. Crows could then modify this template during their extensive experience of foraging with the tool via differential reinforcement^33^, leading to the development of tool innovations. Innovations would then be incorporated into the mental template of subsequent generations allowing for the faithful transmission of tool designs with iterative improvements over time. This hypothesis therefore explains the maintenance of different pandanus tools in the wild over decades, in the absence of specific ecological correlates, and the absence of evidence for imitation in this species. Turning to captivity, the behaviour of the New Caledonian crows tested here also bears considerable similarities to one of the most famous instances of tool manufacture by these birds. In 2002, Betty, a captive New Caledonian crow, spontaneously bent a piece of wire into a hook to pull a bucket out of a tube^34^. Betty had successfully used a pre-made hook to obtain the bucket on a small number of preceding trials; however, in follow-up tasks she did not appear to possess a full causal understanding of hooks^35^. One explanation for this surprising behaviour is that Betty had formed a mental template of a hooked wire, which she then reproduced.

The mental template matching hypothesis also fits with our current understanding of avian song learning. The acquisition of birdsong comprises a memorisation phase, during which a juvenile acquires a song template from listening to conspecifics, followed by a production phase, during which juveniles adjust their own vocalisations until they match that of the memorised template^29^. Although some researchers do not include song in discussions of animal culture (see discussion in^36^) strikingly, song learning – among both birds and cetaceans – is currently the only domain for which there is robust evidence that cumulative cultural evolution does occur among nonhuman animals^37,38^. That is, changes in songs are demonstrably passed among individuals via learning, and these changes can accumulate over time^39,40^. New Caledonian crows are vocal learners, displaying cultural call variation in the wild^41^; thus, these birds possess the neural architecture for memorising and reproducing auditory input^42^. In light of our findings, we hypothesize that a similar mechanism may potentially enable them to memorise and reproduce material artefacts.

One key prediction of the mental template matching hypothesis is that this ability transmits information about tool design with high fidelity. Here, it is important to note that our arbitrary manufacture task likely underestimated the fidelity with which tool designs could be reproduced by wild crows. First, we supplied birds with a novel material: card. Card does not rip in a wholly predictable manner and is likely to be a more challenging material than pandanus leaves for these crows (particularly juveniles) to manipulate accurately with only their beak and feet. Pandanus leaves, in contrast to paper, rip in straight lines due to the veins that run parallel to their leaf edges, and can be snipped into precisely with the beak. Thus, the properties of pandanus leaves, used in the wild, limit variation in the form the tool can take. This may allow for higher-fidelity transmission of natural tool designs, than we observed using card that does not rip in fixed, straight lines. Second, the designs we provided were arbitrary, and birds were rewarded at random for the items they produced. This design choice was necessary in the current experiment to ensure performance during manufacture test trials could not be explained by trial-and-error learning or operant conditioning; however, in the wild, producing functional tools has high adaptive significance^43^, as non-functional deviations from a standard design cannot be used to rake in food. Thus, under natural conditions, it is likely that a capacity for mental template matching would be scaffolded by additional mechanisms, including trial-and-error learning, to facilitate the high-fidelity transmission of tool designs^44,45^. Future work, assessing New Caledonian crow manufacture under conditions that more closely replicate their natural environment is needed to confirm this.

Further research should also consider how long New Caledonian crows’ mental representations persist over time. In our experiment, the delay between reminder trials and manufacturing trials was short, allowing us to confirm that any failures to replicate the templates could not have stemmed from forgetting which template was rewarded. However, in the wild, the delay between using another individual’s tools and manufacturing one’s own is likely to be much greater than the intervals tested here. Understanding more about the nature of these crows’ mental representations – including how this information is stored and for how long – will help us to interpret these birds’ behaviour in the wild.

Whether the cognitive abilities demonstrated here are unique to New Caledonian crows, or are more phylogenetically widespread, is currently unknown. Several species manufacture tools^30,46^ or perform construction behaviours, such as nest building^47^, and may have the opportunity to observe or use end-products made by other individuals. Another corvid species, rooks, do not habitually manufacture tools in the wild, but will in captivity^48^ (as do a small number of other species, such as Goffin cockatoos^49^), suggesting that the cognitive abilities demonstrated here might also be present in related species. Of particular interest is whether some form of mental template matching might account for the transmission of manufactured tool designs among primates, where debate over the existence of cumulative cultures is ongoing^23,50,51^.

To date, emulative learning mechanisms – learning from end-results rather than actions – have been considered by many researchers to be insufficient to support cumulative cultural evolution^8,13^. However, the argument that imitation, teaching and language are the only transmission mechanisms capable of supporting cumulative material cultures may stem in part from the fact that the clearest examples of cumulatively evolved human traditions are cognitively opaque^52^. That is, they involve products for which construction techniques are difficult to infer simply from viewing the product’s final form^53^. This is the case for Acheulean stone tools, where a novice cannot infer the precise technique used to strike a core simply from inspecting a finished tool^12^. However, many situations – including the creation of pandanus tools by New Caledonian crows – are likely to be more cognitively transparent, where manufacture methods can be inferred or discovered without explicit guidance. Here, emulation could be sufficient to enable cultural transmission and evolution. Evidence for this comes from human transmission-chain studies, where end-state emulation can lead to cumulative improvements on cognitively transparent tasks, such as constructing paper planes^18^ or baskets to transport rice^17^, but not on cognitively opaque tasks, such as manufacturing stone tools^54,55^. Prior to the emergence of stone tools, it is likely that hominin tool behaviour involved a greater proportion of cognitively transparent behaviours^56^, and emulative processes may have played an important role in their transmission^17^. These findings also raise the possibility that other cases of transparent tool manufacture, such as the varied fishing probes manufactured by chimpanzees^50^, could potentially allow for cumulative cultural evolution. In sum, our results provide the first demonstration, to our knowledge, that a non-human, tool-making species can manufacture items that match the size of previously rewarded templates. Our findings take the first step towards uncovering why New Caledonian crows show evidence of cumulative cultural evolution. While further work is clearly needed to test other predictions of the mental template matching hypothesis, our results do establish this mechanism as a leading contender for the wild tool designs of this species. A capacity for manufacture via emulation, through a mental template matching mechanism, could potentially reflect one of the minimal cognitive requirements for the emergence of cumulative material cultures.

## Materials and Methods

### Ethics Statement

All aspects of this research were conducted under approval from the University of Auckland ethics committee (reference: R602), and in accordance with ASAB guidelines for animal behavioural research.

### Subjects

Subjects were 8 wild New Caledonian crows, caught and temporarily housed in an 11-cage outdoor aviary on Grande Terre, New Caledonia. Based on sex-size dimorphism 4 birds were female. Based on mouth colouration 4 birds were juveniles less than 2 years old (Blue, Anton, D3R, D4R). Two pilot birds were caught and tested in 2014 (D3R, D4R), and six birds were caught and tested in 2015. All birds were released at their site of capture after testing. These subjects were caught from areas with no obvious pandanus bushes and they did not manufacture tools from pandanus or use provided pandanus strips as tools in the aviary; thus it is unlikely that these particular birds manufactured pandanus tools in the wild.

### Apparatus

The vending machine was a 33 × 30 × 20 cm wooden box with a 6.3 × 3 cm slot in its top surface into which the crow could insert items. Rewards (bottle caps containing meat) were dispensed from an adjacent slot by the experimenter at the push of a button from outside the cage.

### Pre-training

Subjects were first trained to drop stones, then white paper squares (35 × 35 mm, 80GSM paper), into the vending machine to receive rewards. The number of trials taken to acquire stone dropping varied, but all birds inserted at least 24 paper squares at this stage. They then received a spontaneous ripping test. Subjects received 10 × 2-minute trials in which large sheets of white paper (10–15 cm²) were provided, from which they could manufacture items to drop into the vending machine. Half of the birds ripped sections from these sheets without training, the remainder were shaped to rip paper. In shaping trials birds received partially ripped sheets, and the quantity of rips were decreased until the bird would tear sections from unmodified white paper sheets. All manufactured items dropped into the dispenser were rewarded, and birds manufactured and inserted at least 24 pieces of paper at this stage. The birds then experienced that only certain items were rewarded in a colour discrimination test. 6 of 8 birds learnt to drop only a rewarded colour of paper into the vending machine within 30 training blocks, these 6 birds then received a manufacture test. Here, sheets of both the rewarded and unrewarded colours were presented; subjects were rewarded for manufacturing items from the correct colour only. All tested birds, except Blue, passed with at least 19/24 correct choices (binomial test: p < 0.05). Following this, and immediately prior to the experimental training all birds (except the first pilot bird: D3R) were required to manufacture 20 pieces from card (160GSM) as a baseline measure (Supplementary Figure S3).

### Experimental Training Procedure

**Figure 2:**
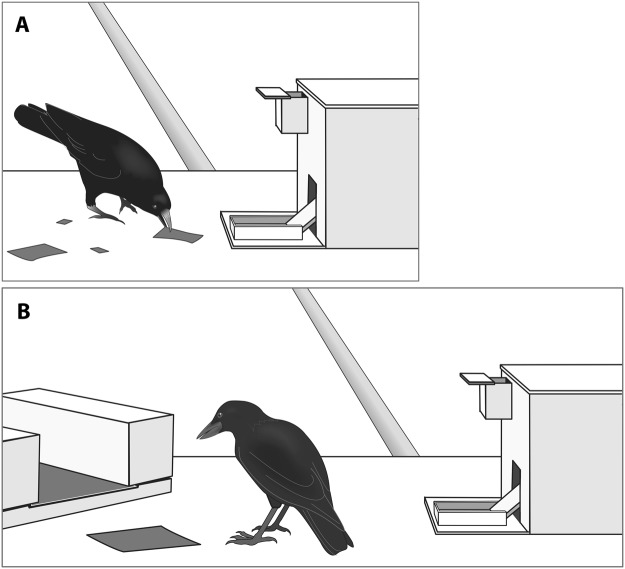
Diagrams of the experimental set up. (**A**) Birds learnt that either small or large templates could be inserted into the vending machine to obtain rewards. (**B**) They then received two very large sheets of card (one presented loose and one fastened under wooden blocks), from which they could manufacture pieces to drop into the vending machine. Credit to Vivian Ward.

To assess whether New Caledonian crows were capable of template matching, birds were trained that either large (40 × 60 mm) or small (15 × 25 mm) pieces of card could be inserted into the vending machine to obtain rewards (Fig. 2A). On each block 8 large and 8 small templates were placed on the table next to the vending machine. Birds could drop these pieces into the vending machine until all 8 of the rewarded size had been inserted and rewarded, at which point the block ended. Training continued until the subject inserted all 8 pieces of the rewarded size and none of the unrewarded size into the vending machine on 5 consecutive blocks (this criterion was set at only 2 consecutive blocks for the pilot birds: D3R & D4R). This training took 2–4 days (11–19 blocks, including criterion blocks) per condition to complete.

### Manufacture Test Procedure

Over the course of the manufacture test trials birds were given the opportunity to manufacture 20 pieces of card to drop into the dispenser. Test trials were conducted in blocks over 1–2 consecutive days, beginning either the same day or the day after training completed. At the start of each block the bird received a reminder trial where 2 large and 2 small pieces were placed on the table and the bird could drop the correct option into the vending machine to receive rewards (Supplementary Video S1, Figure S4). If a bird made a mistake during a reminder trial the test was abandoned and the bird reverted to training trials until they completed one block with no mistakes. This occurred 3 times. Approximately 30–90 seconds after the reminder trial birds began a manufacture trial where they were given two sheets of card with which they could manufacture items: one loose sheet (10 × 10 cm), and one secured under two wooden blocks (accessible section: 21 × 16 cm, Fig. 2B). The loose sheet was too large to fit into the slot in the vending machine without being torn. No templates were present during manufacture trials. Subjects were allowed to rip up either piece of card and insert the ripped pieces into the vending machine. Subjects were rewarded on 50% of trials, regardless of the size of the piece they inserted. To maintain motivation all subjects (except the first pilot bird: D3R) were permitted to rip multiple pieces per trial, with birds manufacturing an average of 1.97 pieces per trial (range: 1–6). The experimenter attempted to enter the room and end the trial after 2 pieces had been manufactured, but did not interrupt if the bird rapidly began manufacturing another item. Each block comprised 3 reminder trials, alternating with 2 manufacture trials, and a maximum of 8 rewards were dispensed on each block. Testing continued until birds made 20 pieces, which took 4–10 blocks per bird. Once birds had completed one size, they were then trained that the alternative size was rewarded using a new colour of card and the manufacture test was repeated. Condition order was counterbalanced across birds.

### Analysis

All sections of paper and card ripped by the birds were collected and scanned. Given the quantity and the non-uniform shapes of the ripped pieces a python script utilizing the openCV computer vision library was employed to measure their area and key dimensions (script accessible from https://figshare.com/s/a6b74be4559712fd05d8). The scanned images were first run through a thresholding algorithm that separated each piece from the background image. They were then processed by a contour finding algorithm, which programmatically determined the borders of each piece. From this information, the area of each piece was calculated, first in pixels, and then translated into millimetres. Two bounding boxes were also calculated. The first specified the maximum and minimum X and Y coordinates of each piece, and the second determined the bounding box of best fit (defined by the rectangle with the lowest surface area) by algorithmically rotating a bounding rectangle around each piece. The length and width of this bounding box were used as an approximation of the length and width of each ripped piece (Supplementary Figure S5).

Statistical analyses were conducted in SPSS v. 21 and R 3.3.0. To determine whether different sized pieces were manufactured in the small and large conditions we fit a linear mixed effect model on area (log-transformed for normality), with the condition (small or large) as a fixed effect and bird as a random effect. Mann-Whitney U-tests determined whether individual birds manufactured differently sized pieces in the small and large conditions. To test for order effects, GLMMs on area (log-transformed for normality) were run with trial order as a fixed effect and bird as a random effect (due to the within-subject nature of our design, trial order was nested within bird).

## Electronic supplementary material

Supplementary Information

supplementary dataset

supplementary video
