# Publisher Correction: Mental template matching is a potential cultural transmission mechanism for New Caledonian crow tool manufacturing traditions

**DOI:** 10.1038/s41598-018-37178-2

**Published:** 2019-03-06

**Authors:** S. A. Jelbert, R. J. Hosking, A. H. Taylor, R. D. Gray

**Affiliations:** 1 Department of Psychology, University of Cambridge, Cambridge, CB2 3EB UK; 2 School of Psychology, University of Auckland, Auckland, 1010 New Zealand; 3 Center for e-Research, University of Auckland, Auckland, 1010 New Zealand; 4 Max Planck Institute for the Science of Human History, Jena, 07745 Germany; 5 Research School of the Social Sciences, Australian National University, Canberra, 2601 Australia

Correction to: *Scientific Reports* 10.1038/s41598-018-27405-1, published online 28 June 2018

Supplementary files containing the Supplementary Dataset containing supporting data and Supplementary Video containing illustrative footage were omitted from the original version of this Article. This has been corrected in the HTML version of the Article; the PDF version was correct at time of publication.

