# Supplementary material for: Mental template matching is a potential cultural transmission mechanism for New Caledonian crow tool manufacturing traditions
Source: Sci Rep. 2018 Jun 28;8:8956. doi: 10.1038/s41598-018-27405-1 (PMC6023922; doi:10.1038/s41598-018-27405-1)
Supplement: Supplementary file 1 — Supplementary Information [file 41598_2018_27405_MOESM1_ESM.pdf]

Supplementary Information for: **Mental template matching is a potential cultural transmission mechanism for New Caledonian crow tool manufacturing traditions.**

Jelbert, S.A., Hosking, R.J. Taylor, A.H. & Gray, R.D.

List of supplementary materials:

- Figures S1, S2, S3, S4 & S5
- Video: Example trials by Emma in the Large and Small conditions.
- Data: Full data set.

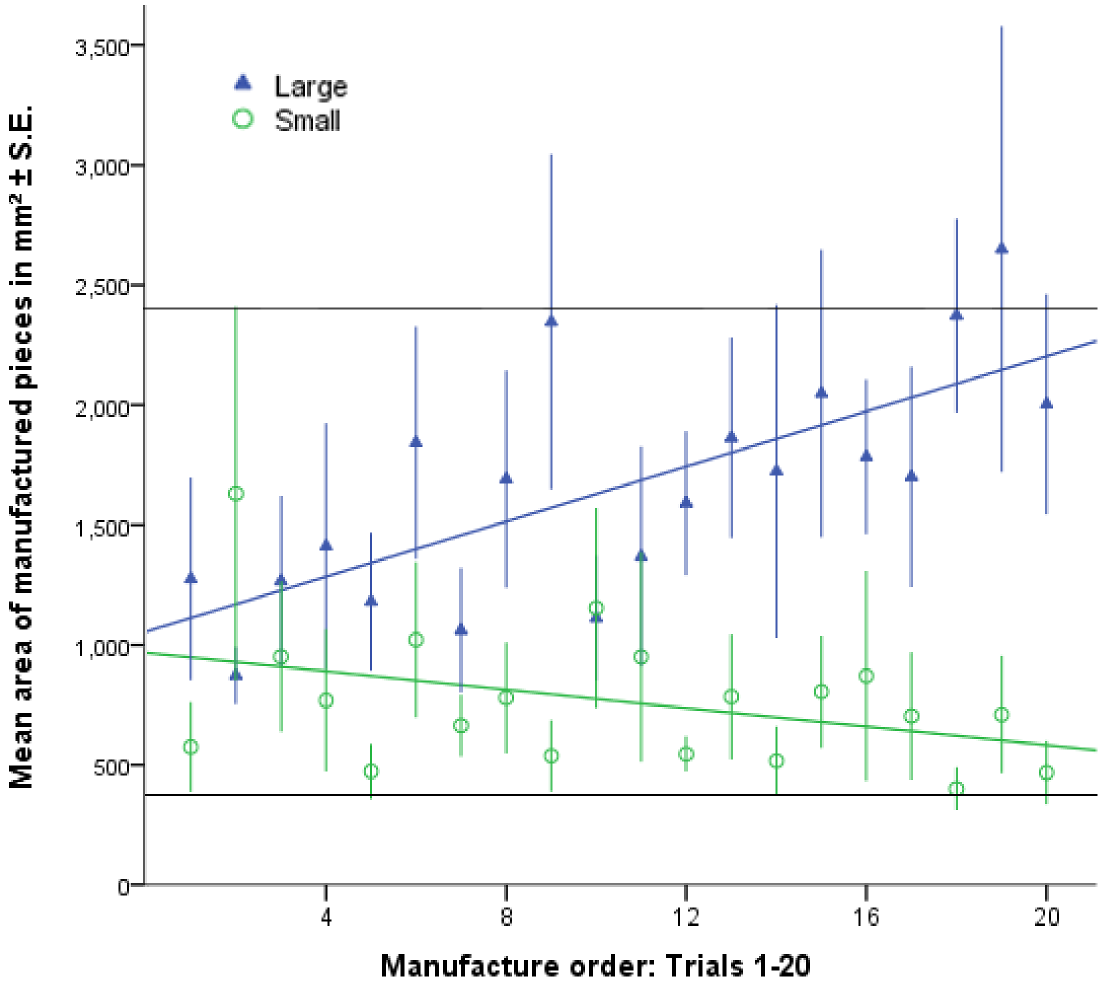

Figure S1. Change in the mean area of manufactured pieces over time. Reference lines indicate the area of the large and small templates. There was a significant difference in the size of manufactured pieces over time in both conditions; in the large condition birds manufactured larger pieces in later trials (GLMM: p=0.0004) and in the small condition they manufactured smaller pieces in later trials (GLMM: p=0.05).

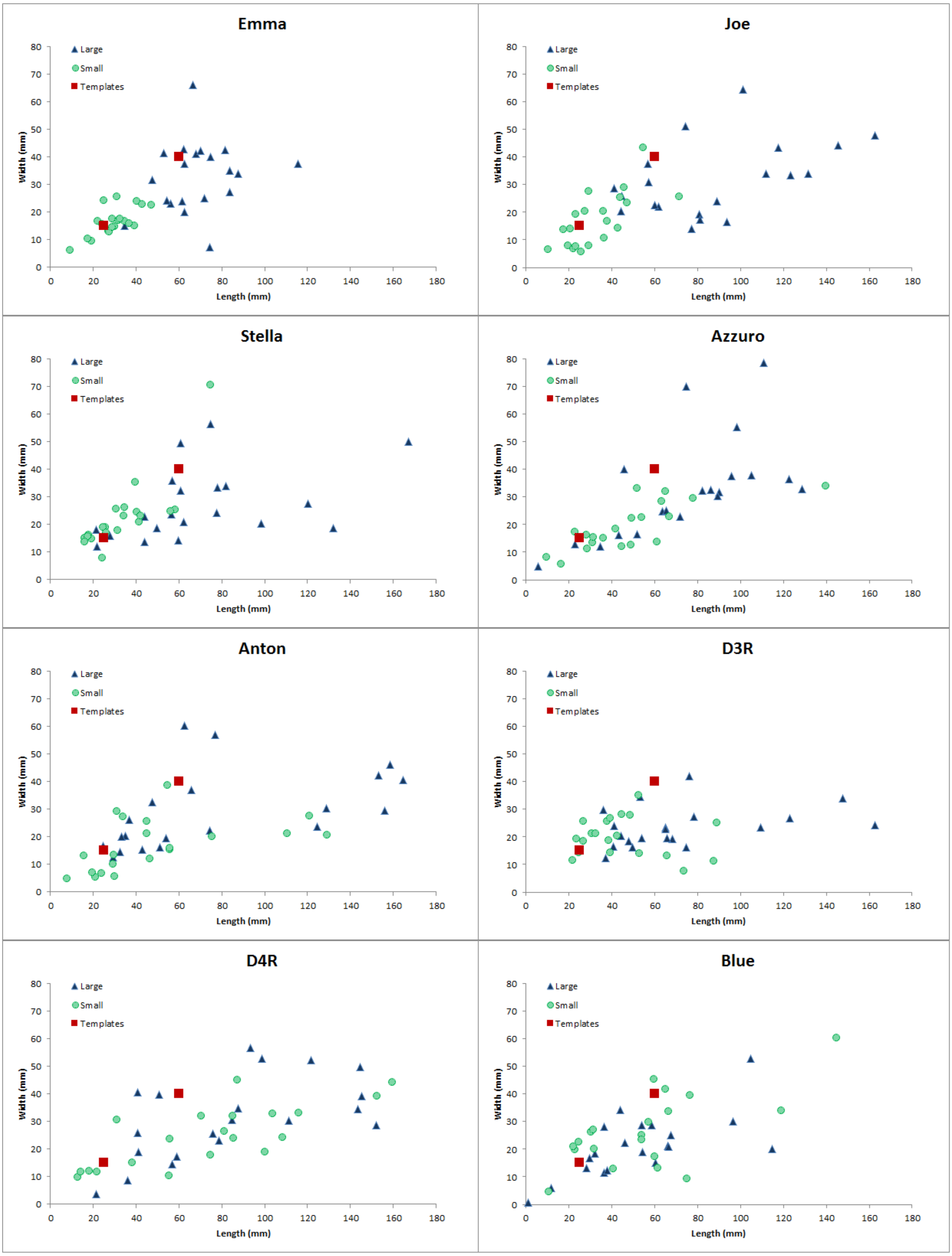

Figure S2. Plots of the length by width for each piece manufactured in the small and large conditions. Template dimensions are provided for comparison (red squares). Lengths and widths were approximated by calculating a bounding box of best fit for each manufactured piece (see Methods).

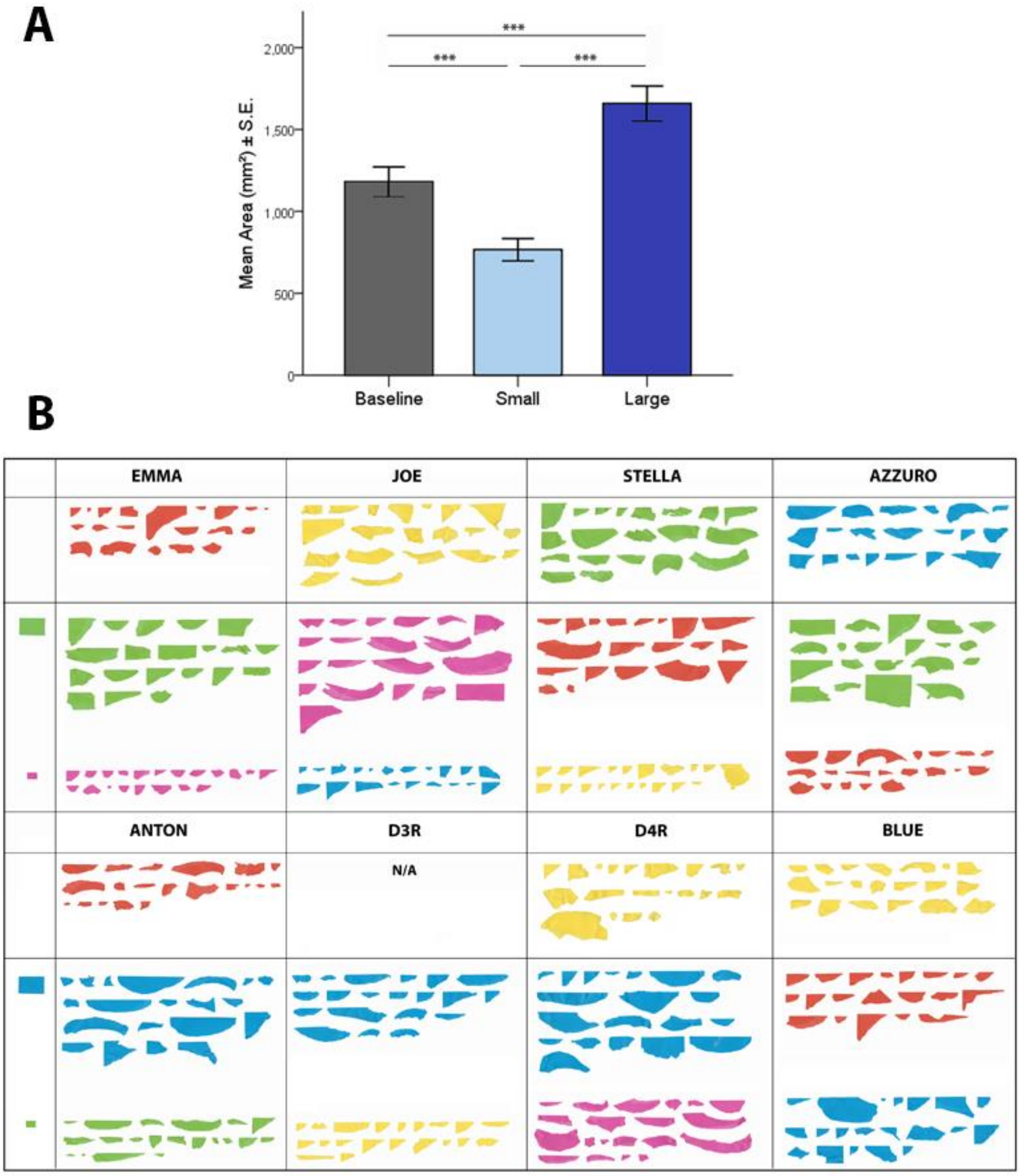

Figure S3. Comparisons to Baseline. All birds, except D3R, manufactured 20 pieces from card (baseline) before being exposed to either of the templates. **A:** The mean area of pieces manufactured in the small and large conditions, compared to baseline. A linear mixed effect model was run on area (log-transformed for normality) with condition (baseline, small, large) as a fixed effect and bird as a random effect. *** pairwise comparisons, $p < .001$. **B:** Scanned images of the pieces manufactured during baseline, presented above those produced in the large and small conditions. Experimental condition order was counterbalanced; Stella, Azzuro, D3R & Blue received the small condition before the large condition.

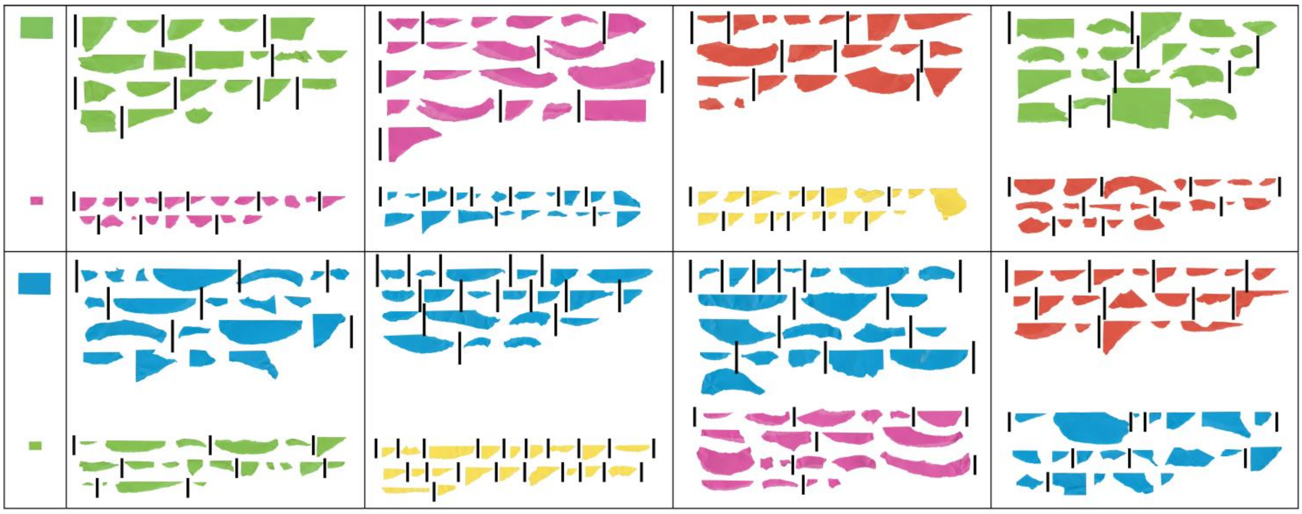

Figure S4. Occurrence of reminder trials within each test. Within test sessions birds were given reminder trials (where large and small templates were provided), followed by manufacture trials (where materials were provided from which birds could manufacture items). Black lines indicate the timing of the reminder trials. Manufactured pieces are presented in the order in which they were manufactured (L-R). The number of pieces made in each manufacture trial varied for two reasons. First, to maintain motivation all subjects (except the first pilot bird: D3R) were permitted to rip multiple pieces per manufacture trial. The experimenter attempted to enter the room and end the trial after 2 pieces had been manufactured, but did not interrupt if the bird rapidly began manufacturing another item. Second, the bird could choose to leave the table after manufacturing one item only. D3R was permitted to manufacture more than one item on a few manufacture trials in her second condition only (Large). The area of the pieces made by the birds immediately after a reminder trial and pieces made later in the test session were not significantly different [LMMs on area, log-transformed for normality, with bird as a fixed factor (excluding D3R). There was no effect of reminder trial order (piece manufactured immediately after a reminder trial: 1, or not: 0) on the log of the area of the pieces made. Type III tests: Large condition: $F\,(6{,}135) = 0.35$, $p = .56$; Small condition: $F\,(6{,}132) = 2.1$, $p = .15$)]. However, note that our study was not specifically designed to test the effect of the timing of reminder trials on the size of manufactured pieces. Thus, the latency between reminder and manufacture trials was not standardised, and, when multiple pieces were made, they were typically manufactured in rapid succession. Each panel represents one bird. From left-right: Emma, Joe, Stella, Azzuro, Anton, D3R, D4R, Blue.

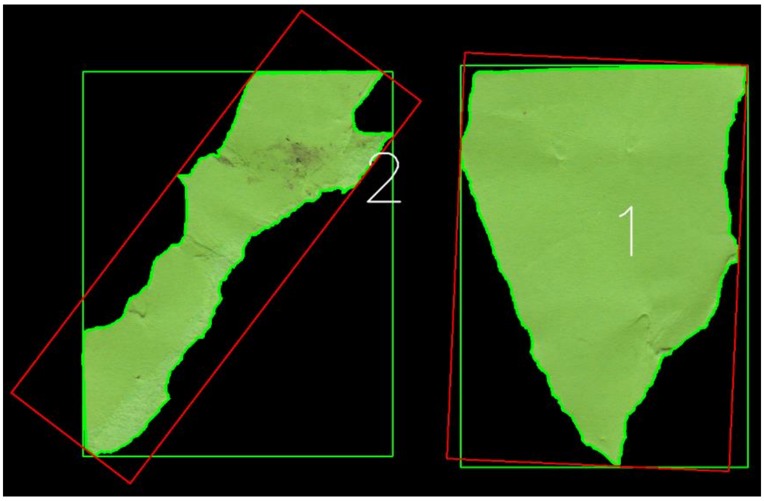

Figure S5. Example processed image with bounding boxes to determine length and width.
